# A simplified herbal decoction attenuates myocardial infarction by regulating macrophage metabolic reprogramming and phenotypic differentiation via modulation of the HIF-1α/PDK1 axis

**DOI:** 10.1186/s13020-024-00933-x

**Published:** 2024-05-30

**Authors:** Zhi-jun Lin, Xin Dong, Huan He, Jia-lin Jiang, Zhuo-ji Guan, Xuan Li, Lu Lu, Huan Li, Yu-sheng Huang, Shao-xiang Xian, Zhong-qi Yang, Zi-xin Chen, Hong-cheng Fang, Ling-jun Wang

**Affiliations:** 1 State Key Laboratory of Traditional Chinese Medicine Syndrome,The First Affiliated Hospital of Guangzhou University of Chinese Medicine, No. 12 Jichang Road, Baiyun District, Guangzhou, 510405 People’s Republic of China; 2 Lingnan Medical Research Center, Guangdong Clinical Researh Academy of Chinese Medicine, No. 12 Jichang Road, Baiyun District, Guangzhou, 510405 People’s Republic of China; 3 Guangzhou Key Laboratory of Chinese Medicine for Prevention and Treatment of Chronic Heart Failure, Guangzhou, 510405 People’s Republic of China; 4 Shenzhen Hospital of Integrated Traditional Chinese and Western Medicine, No. 3 Shajing Street, Bao’an District, Shenzhen, 518104 People’s Republic of China

**Keywords:** Nuanxinkang (NXK), Myocardial Infarction (MI), Energy metabolism, Macrophages polarization

## Abstract

**Background:**

Myocardial infarction (MI) poses a global public health challenge, often associated with elevated mortality rates and a grim prognosis. A crucial aspect of the inflammatory injury and healing process post-MI involves the dynamic differentiation of macrophages. A promising strategy to alleviate myocardial damage after MI is by modulating the inflammatory response and orchestrating the shift from pro-inflammatory (M1) to anti-inflammatory (M2) macrophages, aiming to achieve a reduced M1/M2 ratio. Nuanxinkang (NXK), a simplified herbal decoction, has demonstrated noteworthy cardioprotective, inflammation-regulating, and myocardial energy metabolism-regulating properties.

**Methods:**

In this study, we constructed an MI model by ligating coronary arteries to investigate the efficacy of NXK in improving ventricular remodeling and cardiac function. Mice were administered NXK (1.65 g/kg/d) or an equivalent volume of regular saline via gavage for 28 consecutive days, commencing the day after surgery. Then, we conducted echocardiography to assess the cardiac function, Masson staining to illustrate the extent of myocardial fibrosis, TUNEL staining to reveal myocardial apoptosis, and flow cytometry to analyze the polarization of M1 and M2 macrophages in the hearts. Besides, a lipopolysaccharide (LPS)-induced pro-inflammatory macrophage (M1) polarization model was implemented in RAW264.7 cells to elucidate the underlying mechanism of NXK in regulating macrophage polarization. RAW264.7 cells were pre-treated with or without NXK-containing serum. Oxidative stress was detected by MitoSox staining, followed by Seahorse energy metabolism assay to evaluate alterations in mitochondrial metabolic patterns and ATP production. Both In vivo and in vitro, HIF-1α and PDK1 were detected by fluorescent quantitative PCR and Western blotting.

**Results:**

In vivo, MI mice exhibited a decline in cardiac function, adverse ventricular remodeling, and an increase in glycolysis, coupled with M1-dominant polarization mediated by the HIF-1α/PDK1 axis. Notably, robust responses were evident with high-dose NXK treatment (1.65 g/kg/day), leading to a significant enhancement in cardiac function, inhibition of cardiac remodeling, and partial suppression of macrophage glycolysis and the inflammatory phenotype in MI mice. This effect was achieved through the modulation of the HIF-1α/PDK1 axis. In vitro, elevated levels of mitochondrial ROS production and glycolysis were observed in LPS-induced macrophages. Conversely, treatment with NXK notably reduced the oxidative stress damage induced by LPS and enhanced oxidative phosphorylation (OXPHOS). Furthermore, NXK demonstrated the ability to modify the energy metabolism and inflammatory characteristics of macrophages by modulating the HIF-1α/PDK1 axis. The influence of NXK on this axis was partially counteracted by the HIF-1α agonist DMOG. And NXK downregulated PDK1 expression, curtailed glycolysis, and reversed LPS-induced M1 polarization in macrophages, similar to the PDK1 inhibitor DCA.

**Conclusion:**

In conclusion, NXK protects against MI-induced cardiac remodeling by inducing metabolic reprogramming and phenotypic differentiation of macrophages, achieved through the modulation of the HIF-1α/PDK1 axis. This provides a novel and promising strategy for the treatment of MI.

**Supplementary Information:**

The online version contains supplementary material available at 10.1186/s13020-024-00933-x.

## Highlights

- Balancing the coordination between M1 and M2 macrophages emerges as a promising therapeutic strategy for addressing myocardial infarction.
- Metabolic reprogramming plays a crucial role in the activation and differentiation of macrophages.
- NXK formulation intricately regulates macrophage polarization through the HIF-1α/PDK1 axis within a metabolic-inflammatory network.

**Supplementary Information:**

The online version contains supplementary material available at 10.1186/s13020-024-00933-x.

## Introduction

Myocardial infarction (MI) stands as a paramount contributor to cardiovascular morbidity and mortality, driven by a complex interplay of various pathological processes. Over the last decade, accumulating evidence has highlighted the substantial role of disorders in mitochondrial energy metabolism as the foundation for a wide spectrum of cardiovascular diseases.

From a clinical perspective, MI typically initiates ventricular remodeling (VR), a complex process involving cardiomyocyte changes, fibroblast proliferation, and macrophage infiltration [5]. Following MI, bone marrow-derived monocytes are recruited to the infarcted area and mature into macrophages. Macrophages can be classified into classically activated macrophages (M1) associated with inflammatory responses and alternatively activated macrophages (M2) linked to regeneration and repair [18, 28]. Orchestrating the balance between M1 and M2 macrophages is proposed to provide therapeutic benefits in preventing VR and supporting post-MI recovery [14, 19, 21].

Macrophage activation is intricately regulated, involving signal cascades and epigenetic programming. One key aspect is the distinct bioenergetic requirements of M1 and M2 macrophages, leading to different metabolic profiles in response to the mitochondrial microenvironment cues [12]. Recent studies reveals that M1 macrophages undergo a significant metabolic shift towards glycolysis, disrupting the tricarboxylic acid (TCA) cycle by reducing isocitrate dehydrogenase (IDH) and succinate dehydrogenase (SDH) activity [23]. This results in citrate and succinate accumulation, triggering inflammatory pathways [9]. In contrast, M2 macrophages maintain an intact TCA cycle, facilitating oxidative phosphorylation (OXPHOS). These metabolic differences underscore the significance of metabolic reprogramming in macrophage activation and differentiation.

After MI, the infarcted region becomes ischemic and hypoxic, leading to a reduced oxygen supply for the monocyte-derived macrophages recruited to this zone. Hypoxia-inducible factor-1α (HIF-1α) triggers the expression of pyruvate dehydrogenase kinase 1 (PDK1), initiating cellular glucose metabolism reprogramming, known as the Pasteur effect [24]. This adaptation is vital for macrophage mobilization, as the HIF-1α/PDK1 axis-induced glycolysis drives macrophage migration and activation, particularly during systemic inflammation [17]. This metabolic shift aligns with the observed pattern during the inflammatory injury phase of MI. Heightened glycolysis induced by the HIF-1α/PDK1 axis is proposed to contribute to macrophage migration and significant M1 activation during MI-triggered inflammation.

Nuanxinxinkang (NXK) is a simplified formula composed of *Ilex pubescens* and *radix ginseng Rubra*. Clinical trials [30] and fundamental investigations [3] emphasize its extensive cardioprotective effects, encompassing the restoration of damaged cardiac structure and function, reduction of inflammatory damage, and reprogramming of cardiac energy metabolism. *Radix ginseng Rubra*, acknowledged for its Qi-invigorating properties, enhances immunity and exhibits cardioprotective effects by modulating ACE activity, reinforcing antioxidant defenses, mitigating ROS-induced damage, and preventing cardiac remodeling [1, 13, 20, 22]. *Ilex pubescens* possesses properties such as clearing heat, inflammation, detoxification, activating blood circulation, dispelling stasis, and inducing diuresis, effectively safeguarding the cardiovascular and cerebrovascular system against inflammation [10, 15, 25]. Both ingredients exert significant cardioprotective, anti-inflammatory, and anti-oxidative effects, potentially influencing inflammation regulation post-MI by modulating inflammatory cell metabolism. Despite similarities to classical cardiovascular drugs, the precise targets and molecular mechanisms of NXK remain unknown [3].

In this study, we conducted in vivo and in vitro experiments to investigate whether NXK plays a role in altering macrophage metabolic patterns and regulating polarization phenotypes via the HIF-1α/PDK1 axis. Our goal was to elucidate how NXK contributes to modulating the inflammatory "damage-repair" network in the injured myocardium post-MI, aiming to prevent adverse VR. We anticipate that our research will reveal specific biochemical target factors and the precise molecular mechanisms underlying NXK's effects on modulating macrophage differentiation through metabolic profile shifts. This exploration holds promise for offering a therapeutic approach to treating MI.

## Materials and methods

### Animal models and drug administration

30 SPF-grade C57BL/6J male mice, aged 8 weeks and weighing 22–26 g, were purchased from Guangdong Medical Laboratory Animal Center and housed in the SPF-level Experimental Animal Center of Lingnan Medical Research Center. After 1 week of acclimatization feeding, the mice were used in the following experiments.

A MI mouse model was established by ligating the left anterior descending coronary artery (LAD). Briefly, anesthetized mice were secured in the supine position on a thermostatic operating table following airway ventilation. The ventilator was set with a respiratory rate of 120 breaths/min and a tidal volume of 2.5 mL. The thoracic cavity of the mouse was incised along the left 3rd and 4th intercostal space of the sternum to expose the heart. The LAD was ligated 1–1.5 mm below the left atrium using 8/0 nylon sutures. The SHAM group underwent the same thoracotomy operation without ligating the LAD. Mice were used for experiments 4 weeks after surgery.

NXK used in this study is concentrated NXK powder diluted in sterile water for intragastric administration. The NXK powder comprises only two herbs: *Ilex pubescens* and *radix ginseng Rubra*, with a ratio of 23:77. The concentrated NXK powders were manufactured by the Sci-tech Industrial Park, Guangzhou University of Chinese Medicine, in accordance with internationally certified Good Manufacturing Practice Guidelines (Serial Number: TA2017014). The doses gradient from high to low of NXK are 1.65 g/kg/day (NXK-H), 0.83 g/kg/day (NXK-M), and 0.42 g/kg/day (NXK-L) (The gradient setting referred to the Methodology of Pharmacological Research of Traditional Chinese Medicine). Perindopril (ACERTIL, No. 2016837) served as a positive control drug, and the intragastric dose of perindopril in mice was 0.607 mg/kg/day, calculated according to the recommended daily oral dosage for humans and converted using the equivalent dose formula based on the body surface area of humans and mice. Correspondingly, mice in our study were randomly divided into 6 groups: SHAM group, MI group, NXK-L group (the low dose of NXK), NXK-M group (the medium dose of NXK), NXK-H group (the high dose of NXK, also known as NXK group) and Perindopril group. Mice were given NXK and Perindopril in the corresponding group, while the SHAM group and MI group received equivalent volumes of normal saline water. The drug administration in all groups lasted for 4 weeks consecutively.

The Ethic Committee of Guangzhou University of Chinese Medicine has approved the ethical and scientific application of all animal experiments in our study. The study was strictly conducted in accordance with “Guide for the care and use of Laboratory animals” published by the National Institute of Health.

### Preparation of NXK-containing rat serum

The serum was obtained from 12 SPF-grade SD rats, with an equal distribution of males and females, each weighing 200 g, sourced from the Animal Experiment Center of Guangzhou University of Chinese Medicine. The drug-containing serum was prepared with reference to a recent study [7]. The drug dosage was 1.15 g dry extract powder/kg, which was diluted with sterile water, thoroughly shaken for full dissolution, and appropriately heated before gavage. The dosage is based on the "conversion of the initial dose to equivalent dose" method in the Experimental Methodology of Traditional Chinese Medicine Pharmacology (60 kg for human adults). SPF-grade SD rats, with 3 rats for each gender, were orally gavaged with 1 mL each time, twice a day for five consecutive days. The remaining 6 rats received an equal volume of saline as the control group. Within 3 h after the last gavage, blood was collected from the abdominal aorta, and the serum of the same group was mixed and inactivated in a water bath at 56 °C, then stored at − 80 °C for backup.

### Cell culture and treatment

RAW264.7 cells were cultured in a completed medium composed of 89% 1640 medium (Cat. No. C11875500BT, GIBCO), 10% fetal bovine serum (Cat. No. 10100147, GIBCO) and 1% penicillin/streptomycin (Cat. No.15140122, GIBCO). A pro-inflammatory macrophage (M1) model was established by adding 200 ug/mL lipopolysaccharide (LPS) (Cat.No.L2880-10 mg, Sigma). The specific groupings are as follows: Control group (CTRL): Cells were cultured with medium containing 5% ctrl rat serum + 5% FBS, NXK group (NXK): Cells were cultured with medium containing 5% NXK-containing rat serum + 5% FBS, LPS-induced M1 macrophage model group (CTRL + LPS): Cells were cultured with medium containing 5% ctrl rat serum + 5% FBS with the addition of 200 ug/mL LPS treatment for more than 16 h, NXK treatment of LPS induced M1 macrophage group (NXK + LPS): Cells were cultured with medium containing 5% NXK-containing rat serum and 5% FBS with the addition of 200ug/mL LPS treatment for more than 16 h, DCA (Cat. No. S8615, Selleck) treatment of LPS induced M1 macrophage group (DCA + LPS): Cells were cultured with medium containing 5% ctrl rat serum + 5% FBS with the addition of 200 µg/mL LPS and 100 μΜ DCA treatment for more than 16 h, DMOG (Cat. No. S7483, Selleck) treatment of LPS induced M1 macrophage group (DMOG + LPS): Cells were cultured with medium containing 5% ctrl rat serum + 5% FBS with the addition of 200 µg/mL LPS and 5 mM DMOG treatment for more than 16 h, DMOG and NXK treatment of LPS induced M1 macrophage group (NXK + DMOG + LPS): Cells were cultured with medium containing 5% NXK-containing rat serum and 5% FBS with the addition of 200 ug/mL LPS and 5 mM DMOG treatment for more than 16 h.

### Echocardiography

Four weeks after surgery, the mice underwent examination for cardiac morphology and function using the Vevo 2100 imaging system (Vevo TM 2100). The cardiac functional parameters collected included: left ventricular ejection fraction (LVEF), left ventricular fraction Shortening (LVFS), stroke volume (SV), Left ventricular anterior wall systolic thickness (LVAWs), left ventricular anterior wall diastolic thickness (LVAWd), left ventricular posterior wall systolic thickness (LVPWs), left ventricular posterior wall diastolic thickness (LVPWd), left ventricular end-systolic volume (LVs) and left ventricular end-diastolic volume (LVd).

### Masson’s trichrome staining

Briefly, mouse hearts were excised, and fixed in 4% paraformaldehyde (Art. No. BL539A, Biosharp), embedded in paraffin, and cut into 5 μm thick serial sections. Masson's trichrome staining was performed on each section, and the stained sections were observed using a Pannoramic 250/MIDI microscope (3D HISTECH, Hungary). Image J software was used to measure the fibrotic area in the sections.

### 2,3,5-triphenyl tetrazolium chloride (TTC) staining

After the mice were sacrificed, the heart tissues were collected and briefly rinsed in icy PBS solution to remove residual blood from the ventricles. Then the heart tissues were semifreezed in a − 20 ℃ freezer for 30 min and then cut into 5 slices (1–1.5 mm thick) in a perpendicular way to the long axis. The heart tissue was totally soaked in 1% TTC solution, incubated in a water bath at 37 ℃, stained in the dark for 30 min, and finally fixed with 4% paraformaldehyde and photographed.

### Terminal deoxynucleotidyl transferase dUTP nick end labeling (TUNEL) assay

TUNEL detection was performed using the colorimetric TUNEL Apoptosis Detection Kit (Cat.No.C1091, Beyotime) following the provided instructions. Mouse heart tissue underwent a series of steps: incubation in 4% paraformaldehyde for 24 h, dehydration in 30% sucrose for 24 h, embedding in optimal cutting temperature (OTC) liquid, and slicing into 5 μm thick sections. The sections were immersed in 4% paraformaldehyde for 15 min to immobilize the cells, which was added with immuno-staining strong permeation solution for incubation at room temperature for 5 min, followed by PBS washing, followed by the addition of endogenous peroxidase blocking solution for incubation at room temperature for 20 min, PBS washing, the addition of an appropriate amount of biotin labeling solution for incubation at 37 ℃ for 60 min in the dark, PBS washing, the addition of labeling reaction stopping solution for incubation at room temperature for 10 min, and PBS washing. Subsequently, the Streptavidin-HRP working solution and DAB developing solution were added in sequence. Finally, the slides were sealed with an anti-fluorescence quencher containing DAPI and observed, and images were collected under a fluorescence microscope (Model No. IX73, Olympus).

### Flow cytometric analysis of cellular reactive oxygen species (ROS) and different types of macrophages

Cellular ROS levels were detected using the MitoSox Red Fluorescent Staining Kit (Cat. No. M36008, Thermo), and the working solution was prepared by adding MitoSox Red stock solution to HBSS (containing calcium and magnesium ions) (Cat. No. H1025, Solarbio) at a ratio of 1:1000. The MitoSox Red working solution was incubated with the cells in the dark at 37 °C for 10 min. After incubation, cells were collected, centrifuged at 1000 rpm for 5 min, and the supernatant was aspirated, and the cells were resuspended in 500 µl HBSS.

Flow cytometric analysis of macrophage types in heart tissue: Hearts were immediately excised, flushed with HBSS, and torn into small pieces. Collagenase II (Cat. No. A004174-001, Diamond) was added to HBSS, and heart tissue was digested and ground at 37 °C. The supernatant was collected, erythrocytes were removed using Red Blood Cell Lysis Buffer (Cat. No. BL503B, Biosharp), and then the residue was filtered through a 70 μm filter (Cat. No. 352340, BD). The filtrate was centrifuged (1700 rpm, 5 min) and resuspended in 500 μL of icy HBSS. CD11b (Cat. No. 557672, BD), F4/80 (Cat. No. 565410, BD), Ly6G (Cat. No. 127618, Biolegend), and Ly6C (Cat. No. 128015, Biolegend) antibodies were added to the cell suspension and incubated for 30 min on ice in the dark.

FACS was performed by a MoFlo Astrios EQ (Beckman Coulter) and a FACS Calibur flow cytometer (BD). Data analysis was then performed with FlowJo 10.1 software (Tree Star).

### Quantitative real-time PCR (qPCR) analysis

Total RNA was extracted by RNAzol RT (Cat. No. RN190, Molecular Research Center) in accordance with the manufacturer's instructions. Subsequently, the extracted total RNA underwent reverse transcription, following the guidelines outlined in the Fastking RT reverse transcription kit (Cat. No. KR116, TIANGEN). The resulting cDNA was utilized for quantitative PCR on a CFX96 Real-Time System (Bio-Rad, Hercules) with the SYBR Select master mix kit (Cat. No.4472908, Life Technology). The relative mRNA expression leves of specific genes was normalized to GAPDH, and data analysis was performed utilizing the ΔΔCt method. The primers sequences used for qPCR can be found in Table 1.

**Table 1:** Primer sequences of genes

Gene	Primer sequences
GAPDH	Forward 5′-CATCACTGCCACCCAGAAGACTG-3′ Reverse 5′-ATGCCAGTGAGCTTCCCGTTCAG-3′
ANP	Forward 5′-TATCAGCACCTTGGAGCCGTTG-3′ Reverse 5′-CTCTCGGTCATAGCCATCCAGA-3′
BNP	Forward 5′-TCCTAGCCAGTCTCCAGAGCAA-3′ Reverse 5′-GGTCCTTCAAGAGCTGTCTCTG-3′
COL1	Forward 5′-CCTCAGGGTATTGCTGGACAAC-3′ Reverse 5′-CAGAAGGACCTTGTTTGCCAGG-3′
COL3	Forward 5′-GACCAAAAGGTGATGCTGGACAG-3′ Reverse 5′-CAAGACCTCGTGCTCCAGTTAG-3′
CD86	Forward 5′-ACGTATTGGAAGGAGATTACAGCT-3′ Reverse 5′-TCTGTCAGCGTTACTATCCCGC-3′
Il-1β	Forward 5′-TGGACCTTCCAGGATGAGGACA-3′ Reverse 5′-GTTCATCTCGGAGCCTGTAGTG-3′
TNF-α	Forward 5′-GGTGCCTATGTCTCAGCCTCTT-3′ Reverse 5′-GCCATAGAACTGATGAGAGGGAG-3′
iNOS	Forward 5′-GAGACAGGGAAGTCTGAAGCAC-3′ Reverse 5′-CCAGCAGTAGTTGCTCCTCTTC-3′
YM1	Forward 5′-TACTCACTTCCACAGGAGCAGG-3′ Reverse 5′-CTCCAGTGTAGCCATCCTTAGG-3′
Retnla	Forward 5′-CTTTGCCTGTGGATCTTGGG-3′ Reverse 5′-GGTCCAGTCAACGAGTAAGC-3′
TGF-β	Forward 5′-TGATACGCCTGAGTGGCTGTCT-3′ Reverse 5′-CACAAGAGCAGTGAGCGCTGAA-3′
CD163	Forward 5′-GGCTAGACGAAGTCATCTGCAC-3′ Reverse 5′-CTTCGTTGGTCAGCCTCAGAGA-3′
HIF-1α	Forward 5′-CCTGCACTGAATCAAGAGGTTGC-3′ Reverse 5′-CCATCAGAAGGACTTGCTGGCT-3′
PDK1	Forward 5′-CCACTGAGGAAGATCGACAGAC-3′ Reverse 5′-AGAGGCGTGATATGGGCAATCC-3′
PDH	Forward 5′-AGTAATCCAGCCTCACTTGATGA-3′ Reverse 5′-AGGTTCCCCACAGATGTCAATA-3′
LDHA	Forward 5′-ACGCAGACAAGGAGCAGTGGAA-3′ Reverse 5′-ATGCTCTCAGCCAAGTCTGCCA-3′

### Enzyme-linked immunosorbent assay (ELISA)

The ELISA kits were employed to assess the concentration of IL-1β (Cat. No. JL18222, J&l biological), IL-6 (Cat. No. JL20268, J&l biological), LDH (Cat. No. RD-RX20588, Henghuibio), cTn-I (Cat. No. RD-RX20716, Henghuibio) and CK-MB (Cat. No. RD-RX28610, Henghuibio) in mouse peripheral blood serum and ROS production in mouse heart tissues (Cat. No. E004, Nanjin Jiancheng Bioengineering Institute). The experimental procedures followed the guidelines provided by the respective kit manufacturers.

### PDH activity detection

The heart tissue was ground using an ice bath homogenizer, followed by centrifugation at 11,000*g* for 10 min at 4 °C. The resulting supernatant was collected, and the optical density (OD) of the sample at 605 nm was measured in accordance with the manufacturer's instructions provided with the PDH activity assay kit (Cat. No. YX-W-B103, Sinobestbio). PDH activity was then calculated based on the weight of the heart.

### Western Blot (WB) analysis

Heart tissue and cellular proteins were extracted with RIPA lysis buffer (Cat. No. P0013B, Beyotime).Subsequently, proteins of varying molecular weights were separated via SDS-PAGE (4–20%) and transferred onto 0.45 μm PVDF membranes (Cat. No. IPVH00010, EMD Millipore). After blocking the membranes with 5% skimmed milk (Cat. No. 9999, CST) for 1 h at room temperature, they were incubated overnight at 4 °C with specific antibodies: GAPDH (1: 1000, Cat. No. 2118, CST), HIF-1α (1: 500, Cat. No. bs-0737R, Bioss), and PDK1 (1: 1000, Cat. No. DF4365, Affinity Bioscience) on a shaker. The following day, secondary antibodies (Cat. No. 7074S, CST) were applied and incubated for 1 h at room temperature. Immunoblotting was visualized using ECL (Cat. No. WBKLS0500, Millipore), and the density of immune-reactive bands was analyzed using Image J software.

### Metabolic analysis

For metabolic analysis, RAW264.7 cells were seeded in XFe 24 well microplates at a density of 2 × 10^6^ cells per well (Cat. No. 102340-100, Agilent Technologies). Oxygen consumption rate (OCR), extracellular acidification rate (ECAR), and real-time ATP rate were respectively measured using the Cell Mito Stress Test kit (Cat. No. 103015-100, Agilent Technologies), Glycolysis Stress Test Kit (Cat. No. 103020-100, Agilent Technologies) and Real-time ATP Rate Assay Kit (Cat. No. 103592-100, Agilent Technologies) following the manufacturer's instructions. The measurements of OCR, ECAR, and real-time ATP rate were conducted using a Seahorse XFe24 Analyzer (Cat. No.S7801B, Agilent Technologies), and the results were analyzed with Wave software version 2.6.0 (Agilent Technologies).

### Statistical analysis

SPSS25.0 statistical software was used for data analysis. All data were described as mean standard deviation (X ± SD).T test was adopted to assess the significance of differences between the two groups. For comparisons among multiple groups, one-way ANOVA was employed, with statistical significance set at *P* < 0.05.

## Results

### NXK alleviates the deterioration of cardiac structure and function after MI

**Fig. 1:**
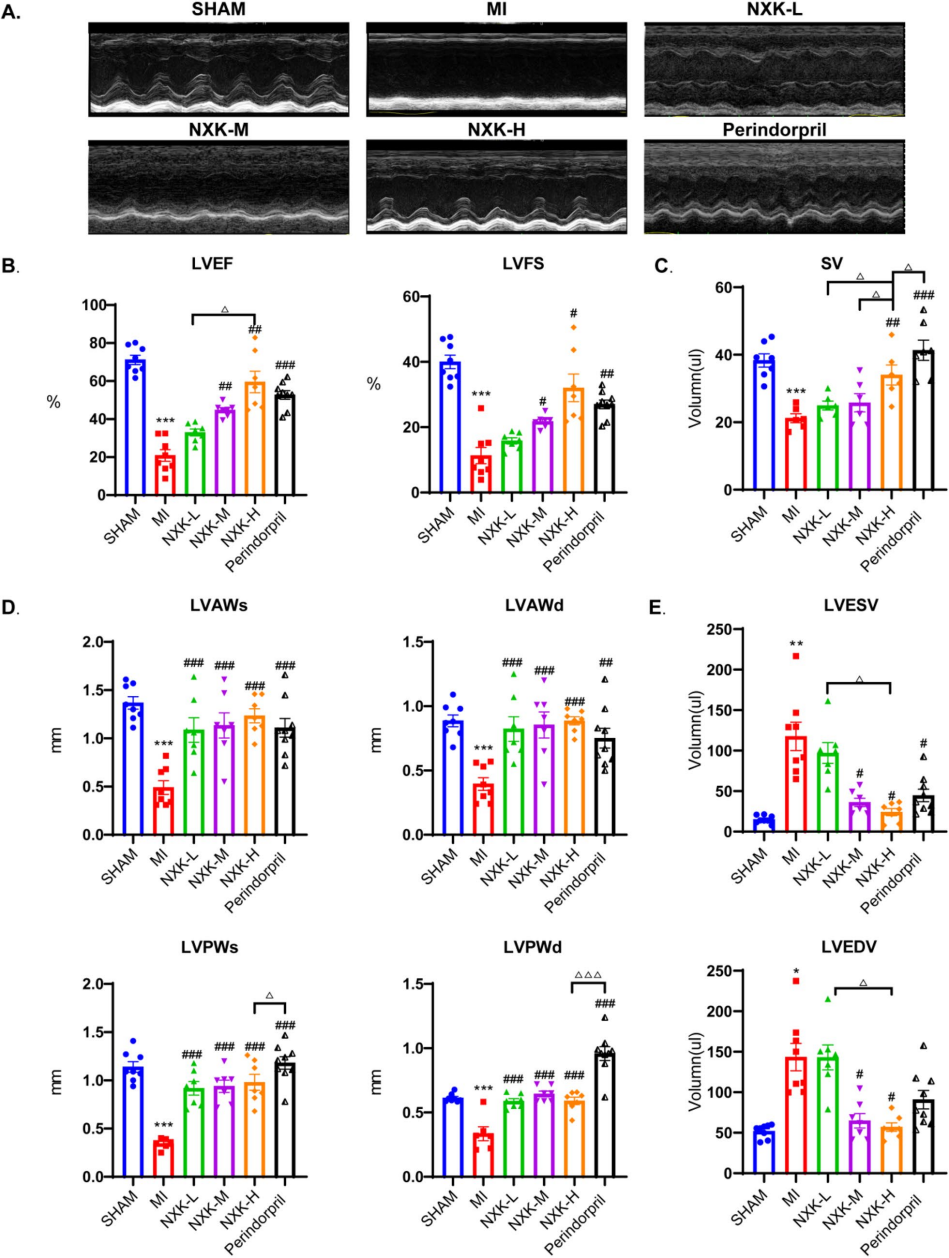
NXK improves cardiac function in MI mice. **A** Representative M-mode echocardiographic images were shown from Sham, MI, MI + NXK groups 28 days post MI. **B**–**E** LVEF, LVFS, SV, LVAWs, LVAWd, LVPWs, LVPWd, LVESV, and LVEDV were measured by echocardiography (n ≥ 6). Values were shown as mean ± SD, ^**^P < 0.01, ^***^P < 0.001 vs the SHAM group, ^#^*P* < 0.05, ^##^*P* < 0.01, ^###^*P* < 0.001 vs MI group. ^△^*P* < 0.05 *vs* the Perindopril group

To evaluate the effect of NXK on cardiac function in the MI model, different dosages (0.42 g/kg/day, 0.83 g/kg/day, and 1.65 g/kg/day) of NXK were administered. Four weeks post-MI, echocardiography was used to assess cardiac function in each group (Fig. 1A). LVEF, LVFS, SV, LVAWs, LVAWd, LVPWs, and LVPWd were significantly lower in the MI group compared to the SHAM group (Fig. 1B–D), whereas LVESV and LVEDV were higher (Fig. 1E). The NXK-M and NXK-H groups exhibited higher LVEF, LVFS, LVAWs, LVAWd, LVPWs and LVPWd, coupled with lower LVESV and LVEDV. However, aside from the improvement in ventricular wall thickness, which mirrored the NXK-M and NXK-H groups, the NXK-L group showed minimal change from the MI group in other cardiac function parameters. Additionally, SV was notably higher in the NXK-H group compared to the NXK-M group, suggesting a dose-dependent improvement in cardiac function with NXK. Moreover, the NXK-H group demonstrated a comparable performance to perindopril in enhancing cardiac function.

**Fig. 2:**
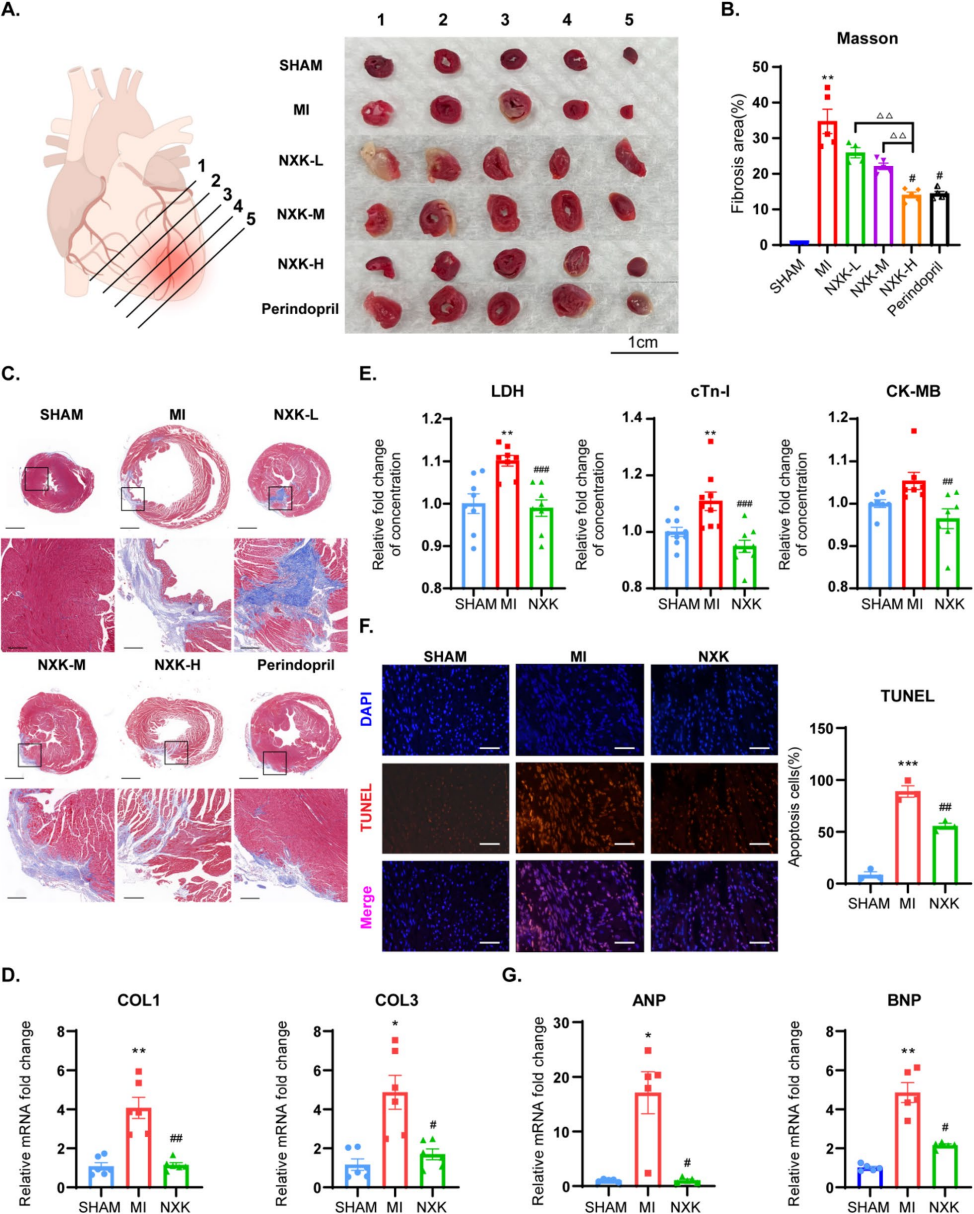
NXK protects against the deterioration of cardiac structure after MI. **A** Representative micrographs depicting TTC staining in various groups. **B** Quantification of fibrosis area from different groups (n ≥ 3). **C** Representative photomicrographs of Masson’s trichrome staining respectively from different groups (upper bars, 1 mm; bottom bars, 200 μm). **D** RT-PCR quantification of COL1 and COL3 mRNA levels in mouse cardiac tissues (n = 6). **E** The concentration of LDH, cTn-I and CK-MB in the serum of mice (n ≥ 7). **F** Representative TUNEL staining (bars, 50 μm) and quantification of positive rate of apoptotic cardiomyocytes in peripheral tissue of infarction (n = 3). **G** Quantification of ANP and BNP mRNA expression (n = 5). **F** TUNEL staining revealed that NXK treatment alleviated cardiac apoptosis in mice. Values were shown as mean ± SD, ^*^P < 0.05, ^**^P < 0.01, ^***^P < 0.001 vs the SHAM group, ^#^*P* < 0.05, ^##^*P* < 0.01 vs the MI group

Apart from the impairment in cardiac function, histological changes in the heart, including adverse cardiac remodeling, are crucial indicators of post-MI cardiac injury. Indeed in LAD-ligation subjected mice, there was marked increase in MI assessed using TTC staining in comparison to the SHAM group, while high doses of NXK and perindopril could relieve myocardial ischemia and rescue damaged cardiomyocytes upon MI injury (Fig. 2A). Masson staining was employed to visualize collagen fiber deposition in heart tissues. MI mice exhibited significantly higher collagen deposition in the infarct area compared to SHAM mice, and treatment with high doses of NXK and perindopril reduced collagen accumulation (Fig. 2B, C). Additionally, as mentioned earlier, mice in the NXK-H group displayed improved cardiac function and reduced collagen deposition. Consequently, the high dose of NXK (1.65 g/kg, referred to as NXK in the following figures) was deemed the most effective and was utilized in subsequent studies. Subsequently, we assessed the mRNA expression levels of COL1 and COL3 through qPCR, revealing that NXK significantly reversed the elevated expression of COL1 and COL3 in the heart 4 weeks after MI (Fig. 2D). LDH, cTn-I, and CK-MB are the biochemical markers of myocardial injury, and their circulating levels are markedly reduced under the treatment of NXK (Fig. 2E). Moreover, TUNEL staining demonstrated that NXK could reduce TUNEL-positive cells, thereby alleviating MI-induced apoptosis in cardiomyocytes (Fig. 2F). Accordingly, NXK treatment exhibited potential in improving cardiac neuroendocrine function post-MI injury, as evidenced by a significant decrease in ANP and BNP (Fig. 2G). The therapeutic effect of NXK on MI maybe due to its effect in reducing myocardial fibrosis and apoptosis after MI, which can protect against ventricular remodeling,

### NXK alleviates oxidative stress and inflammation

**Fig. 3:**
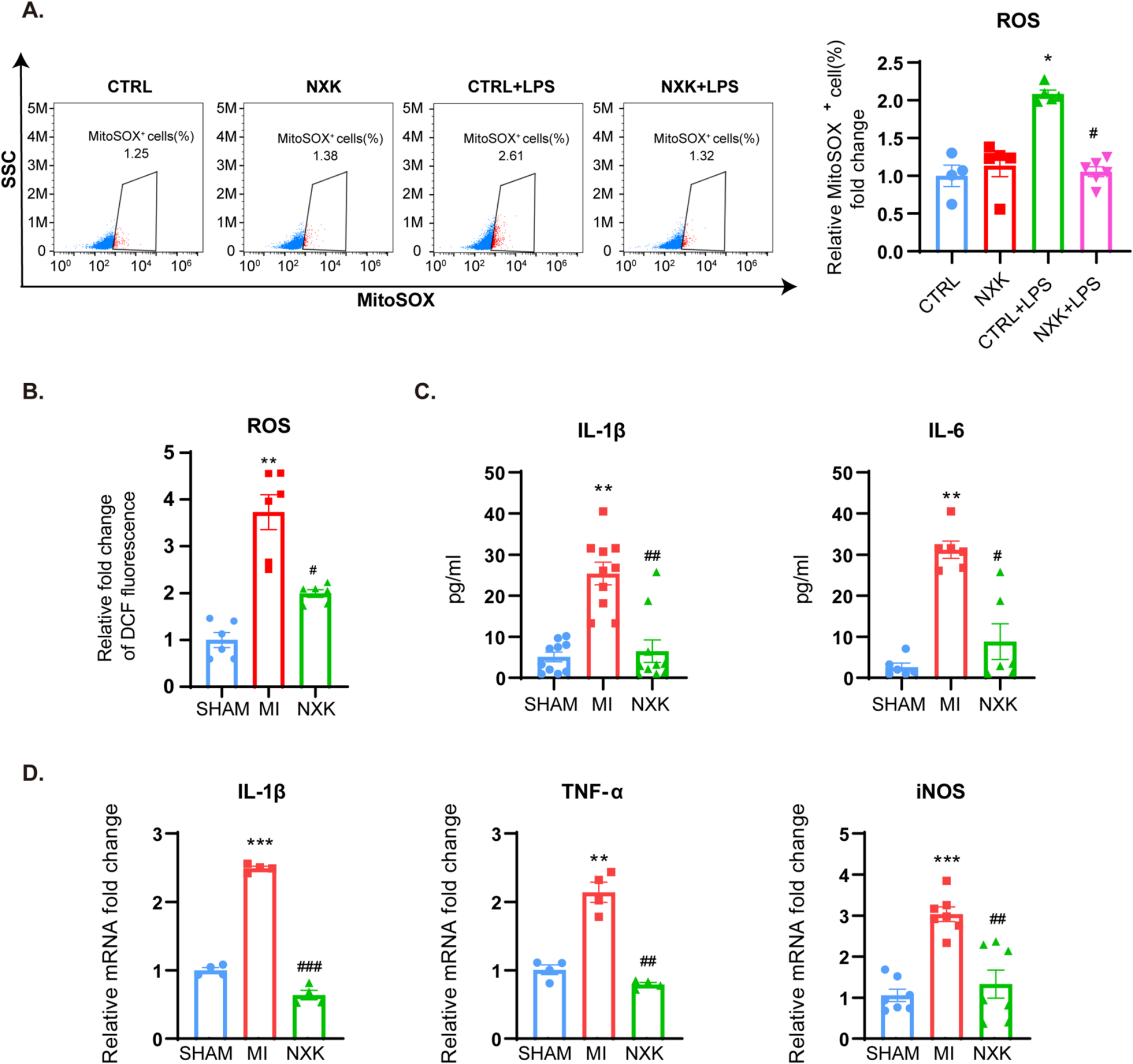
NXK alleviates oxidative stress and inflammatory responses. **A** The representative figure of MitoSOX staining red positive cells in different groups of macrophages. Quantified percent of MitoSOX staining red positive cells (n ≥ 4), all the data were shown as fold change compared with CTRL. **B** Quantification of ROS production in mouse heart tissues (n = 6). **C** The levels of IL-1β and IL-6 antigens in mouse serum were determined by Elisa assays (n ≥ 5). **D** mRNA expression of IL-1β, TNF-α and iNOS were detected by qPCR (n ≥ 4). Values were shown as means ± SD, ^*^P < 0.05, ^**^P < 0.01, ^***^P < 0.001 vs the SHAM group, ^#^*P* < 0.05, ^##^*P* < 0.01, ^###^*P* < 0.001 vs the MI group

To investigate the expression of the pro-inflammatory parameters in RAW264.7 cells cultured with NXK-containing serum after induction of M1 macrophages with LPS in vitro*,* we induced cellular stress in macrophages through LPS stimulation, leading to increased ROS production, as detected by MitoSOX in red fluorescence. Flow cytometry revealed that NXK treatment significantly reduced mitochondrial ROS production compared to LPS-stimulated macrophages (Fig. 3A, Additional file 1: Fig. S1A, B). Similarly, NXK treatment alleviated oxidative stress damage in heart tissue (Fig. 3B). Furthermore, MI injury resulted in elevated serum levels of IL-1β and IL-6 antigens (Fig. 3C) and increased mRNA expression of IL-1β, TNF-α, and iNOS in mouse heart tissue (Fig. 3D). However, intervention with NXK reversed all these upregulated parameters.

### NXK orchestrates the ratio of M1 to M2 macrophages in the heart during the repair phase of MIS

**Fig. 4:**
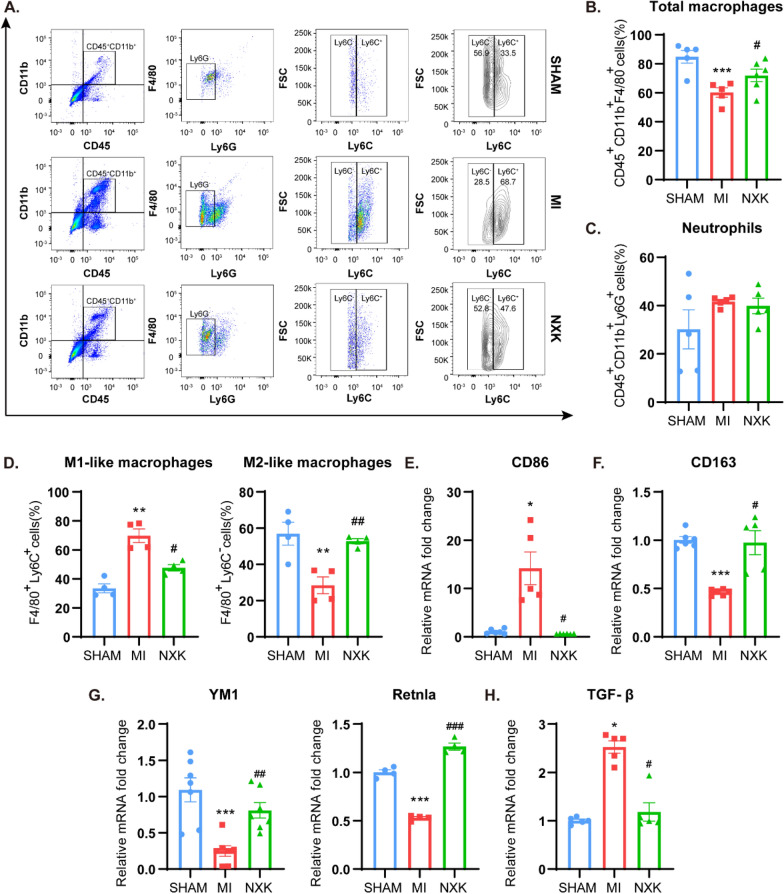
NXK inhibited M1 polarization and promoted M2. **A** Representative flow diagrams of M1-like and M2-like macrophages in different groups. **B**–**D** Quantified percent of total macrophages, neutrophils, M1-like and M2-like macrophages respectively (n ≥ 4). **E**–**H** The mRNA expression of CD86, CD163, YM1, Rentla and TGF-β in vivo (n ≥ 4), were detected by qPCR. Values were shown as means ± SD, ^*^P < 0.05, ^**^P < 0.01, ^***^P < 0.001 vs the SHAM/CTRL group, ^#^*P* < 0.05, ^##^*P* < 0.01, ^###^*P* < 0.001 vs the MI group/CTRL + LPS group

Several studies support the notion that macrophages play an important role in the pathology of inflammation, with their activation and differentiation influencing various immune functions involved in inflammation regulation. MI damages the myocardium, triggering ongoing inflammatory responses. During the inflammatory process, bone marrow-derived monocytes are recruited to the infarction site, differentiating into macrophages. Microenvironmental cues induce macrophages to adopt different phenotypes [16, 27]. During 4 weeks following MI, cardiac infiltration of macrophages was examined by flow cytometry in order to investigate if NXK can affect cardiac repair by controlling macrophage polarization, attenuating cardiac remodeling and inflammation. In heart tissue, CD45^+^CD11b^+^F4/80^+^ cells were identified as macrophages, while CD45^+^CD11b^+^Ly6G^+^ cells were classified as neutrophils. Gating strategy for identifying different cell subsets was adapted from a previous study [4] (Additional file 1: Fig. S1C). Among the macrophages, Ly6C^+^ identified pro-inflammatory M1-like macrophages, and Ly6C^−^ identified M2-like macrophages (Fig. 4A). Following MI surgery, the proportion of total macrophages decreased dramatically (Fig. 4B, Additional file 1: Fig. S1D), whereas the proportion of neutrophils did not differ significantly between groups (Fig. 4C, Additional file 1: Fig. S1E). The proportion of M1-like macrophages increased significantly, while M2-like macrophages decreased. Surprisingly, NXK suppressed M1-like macrophages while significantly increasing M2-like macrophages. NXK also reversed the proportions of total macrophages in MI mouse hearts (Fig. 4D). These findings suggest that NXK may influence macrophage activation and polarization. To assess NXK's impact on macrophage phenotypic differentiation, mRNA expression of M1 and M2 biomarkers was quantified. The mRNA level of CD86, a specific biomarker for M1 macrophages, increased dramatically in response to MI (Fig. 4E), accompanied by elevated levels of the inflammatory cytokines IL-1β, TNF-α, and iNOS (Fig. 3D), indicating M1-like macrophage activation. Conversely, the M2 marker CD163 and M2 cytokines YM1 and Retnla were downregulated post-MI. Unexpectedly, NXK treatment promoted M2 activation and alleviated inflammatory damage induced by M1 overactivity (Fig. 4F, G). Furthermore, M2 overactivation may result in fibrosis due to excessive tissue repair, and moderate secretion of TGF-β by M2 can coordinate extracellular matrix (ECM) homeostasis and promote tissue injury repair, and NXK was found to inhibit the mRNA level of TGF-β (Fig. 4H), suggesting that NXK promotes M2-like macrophage activation within a reasonable range without causing fibrosis. The above findings suggested that NXK inhibits M1 macrophage polarization, coordinate the balance of M1 and M2 macrophages, and aids in controlling inflammation, tissue repair, and mitigating the effects of VR after MI.

### NXK coordinates energy metabolic reprogramming of macrophages

Recent research has highlighted the intricate connection between macrophage metabolic pathways and phenotypic polarization, identifying energy metabolic reprogramming as a key regulatory mechanism in macrophage activation [10, 12, 29]. Generally, ECAR and OCR are distinct markers of the two main energy-producing pathways, OXPHOS and glycolysis. The majority of cells have the ability to shift between these two metabolic states in response to inflammatory stimuli or changes in the tissue microenvironment.

**Fig. 5:**
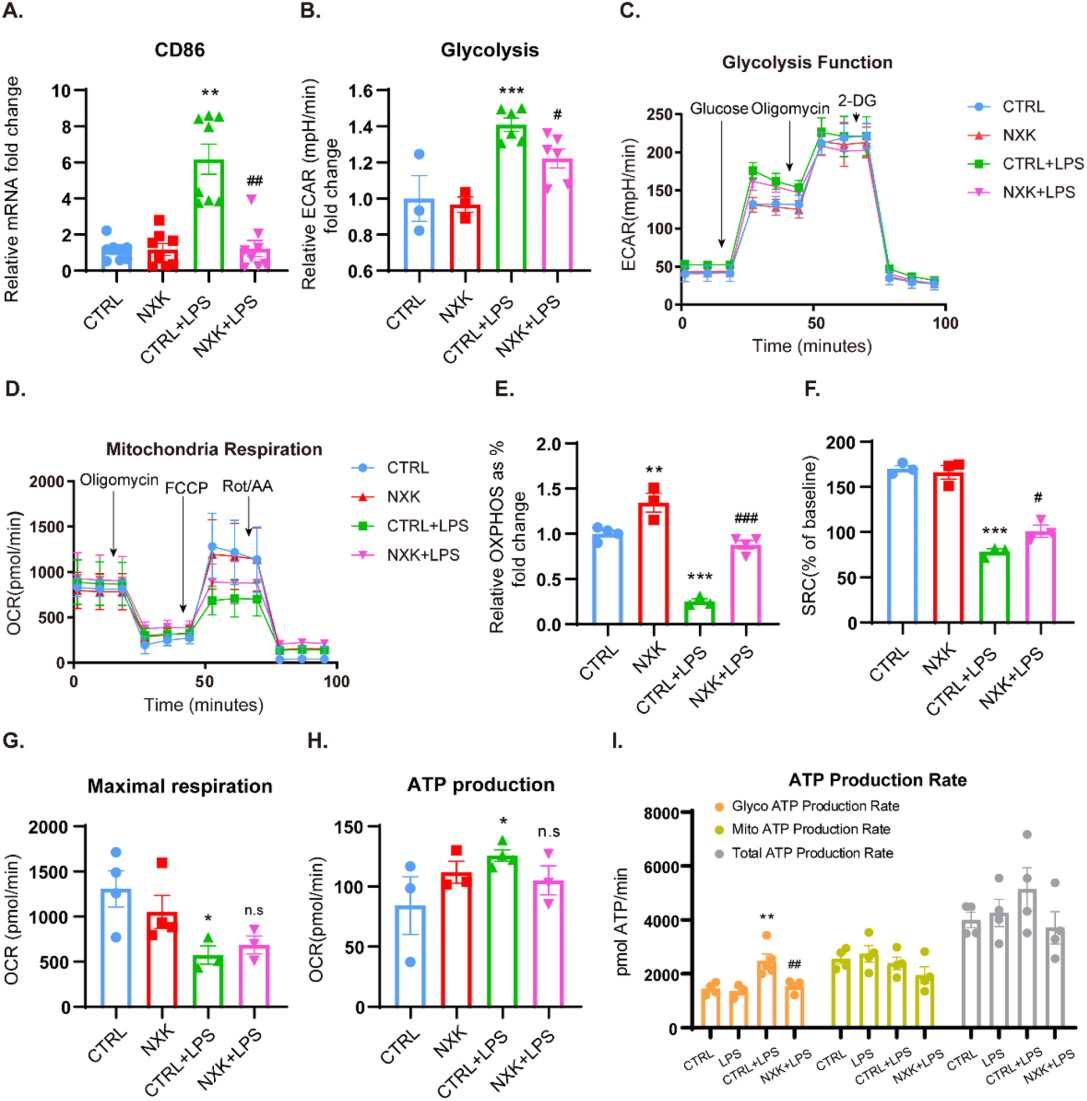
NXK coordinates macrophage polarization via energy metabolism reprogramming. **A** The mRNA expression of CD86 in vitro (n = 8), were detected by qPCR. **B** Glycolysis level were quantitatively analyzed (n ≥ 3). **C** Representative curves of cellular glycolysis function. **D** Representative curves of cellular mitochondrial respiration. **E**–**G** Basal OXPHOS (%), spare respiratory capacity (%), maximal respiration, and ATP production based on mitochondrial aerobic respiration were analyzed quantitatively (n ≥ 3). **I** The real-time ATP production rate and total ATP production level of macrophages were shown (n = 4). Values were shown as means ± SD, ^*^P < 0.05, ^**^P < 0.01, ^***^P < 0.001 vs the CTRL group, ^#^*P* < 0.05, ^##^*P* < 0.01, ^###^*P* < 0.001 vs the CTRL + LPS group, n.s, no significance

To characterize the effect of NXK on macrophage metabolic patterns in response to LPS stimulation, we initially assessed the tendency toward M1-like polarization by evaluating the expression of the M1 macrophage marker CD86 mRNA (Fig. 5A).Subsequently, a glycolysis stress test revealed that LPS stimulation significantly increased ECAR, indicating elevated glycolysis in M1-like macrophages (Fig. 5B, C). Furthermore, the elevated glycolysis rate was accompanied by a rise in glycolytic ATP production (Fig. 5I).Interestingly, NXK treatment reversed the heightened glycolytic parameters, suggesting that NXK reduces glycolysis in LPS-stimulated macrophages. Following that, we examined the mitochondrial respiration and total ATP production rate in each group of cells. Under LPS stimulation, the basal ATP production ratio from mitochondrial OXPHOS to total ATP production, spare respiratory capacity (SRC), and maximal respiration in macrophages were significantly lower than in the control condition (Fig. 5D–G). NXK treatment increased OXPHOS and SRC without compromising ATP production under LPS stimulation (Fig. 5H), indicating that NXK promotes mitochondrial OXPHOS in an energetically balanced manner. Moreover, we compared the energy production efficiency of OXPHOS and glycolysis. While the rate of total ATP production and ATP production from mitochondrial OXPHOS did not significantly differ between groups, NXK treatment notably reduced the rate of glycolytic ATP production (glycoATP production rate) in LPS-stimulated macrophages (Fig. 5I). This suggests that NXK diminishes the energy dependence of LPS-stimulated macrophages on glycolysis. These findings support the notion that during the inflammatory response, macrophages shift their energy production pattern towards glycolysis, and NXK reverses this metabolic pattern to OXPHOS, influencing macrophage phenotype differentiation.

### NXK regulates macrophage energy metabolism via the HIF-1α/PDK1 axis

**Fig. 6:**
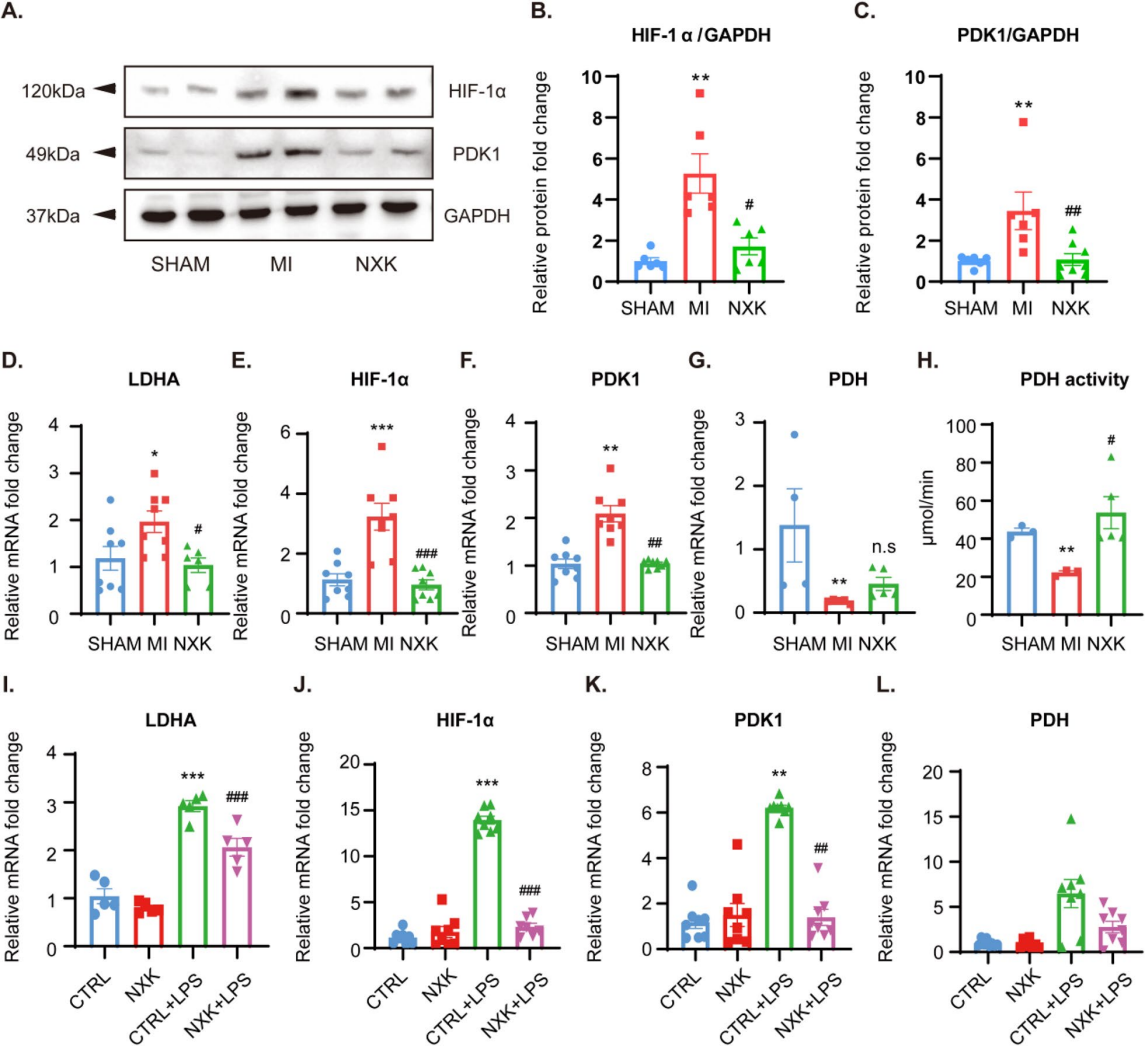
NXK modulates the HIF-1α/PDK1 axis. **A**–**C** Representative WB images and qualified protein expression levels of cardiac HIF-1α and PDK1 (n ≥ 3). **D**–**G** The mRNA expression level of cardiac LDHA, HIF-1α, PDK1, and PDH were detected by qPCR (n ≥ 4). **H** PDH activity was detected by an Elisa kit and analyzed following instructions (n = 3). **I**–**L** The mRNA expression level of cellular LDHA, HIF-1α, PDK1, and PDH were detected by qPCR (n ≥ 3). Values showed as means ± SD, ^*^P < 0.05, ^**^P < 0.01, ^***^P < 0.001 vs the MI group/the CTRL group, ^#^*P* < 0.05, ^##^*P* < 0.05, ^###^*P* < 0.001 vs the MI group/the CTRL + LPS group, n.s, no significance

The active glycolysis induced by the HIF-1α/PDK1 axis is critical for macrophage migratory capacity. HIF-1α is implicated in macrophage migration and differentiation through PDK1-induced glycolysis. This involves the inactivation of the "gatekeeper enzyme" pyruvate dehydrogenase (PDH) via PDK1 phosphorylation, which restricts pyruvate influx into the tricarboxylic acid (TCA) cycle, actively inhibiting mitochondrial OXPHOS [17]. LDHA, responsible for converting pyruvate to lactate during glycolysis, is considered a key marker of glycolysis [26]. Building on these insights, we conducted WB and qPCR to assess the expression levels of key factors in the HIF-1α/PDK1 axis in vivo, aiming to validate the mechanism of this axis in MI. The results showed that NXK treatment reduced the transcription and protein levels of HIF-1α and PDK1 in vivo (Fig. 6A–C, E, F). Additionally, NXK inhibited the expression of the glycolytic gene LDHA (Fig. 6D) and relieved the suppression on PDH activity (Fig. 6H). Notably, PDH mRNA expression remained unchanged (Fig. 6G), indicating that the activity of the HIF-1α/PDK1 axis was significantly heightened after MI. Conversely, NXK demonstrated the ability to reverse glycolysis overactivation, downregulate the expression of this axis, and release PDH enzyme activity.

To further affirm the mechanism of HIF-1α/PDK1 axis, we evaluated the mRNA expression of LDHA, HIF-1α, PDK1, and PDH in vitro. LPS-treated macrophages exhibited an activated HIF-1/PDK1 axis, leading to a significant upregulation of glycolysis. However, when treated with NXK-containing serum, the mRNA levels of LDHA, HIF-1α, and PDK1 were reduced (Fig. 6I–K), while PDH gene expression remained largely unchanged (Fig. 6L), indicating that NXK may regulate metabolic reprogramming of macrophages through modulation of the HIF-1α/PDK1 axis.

**Fig. 7:**
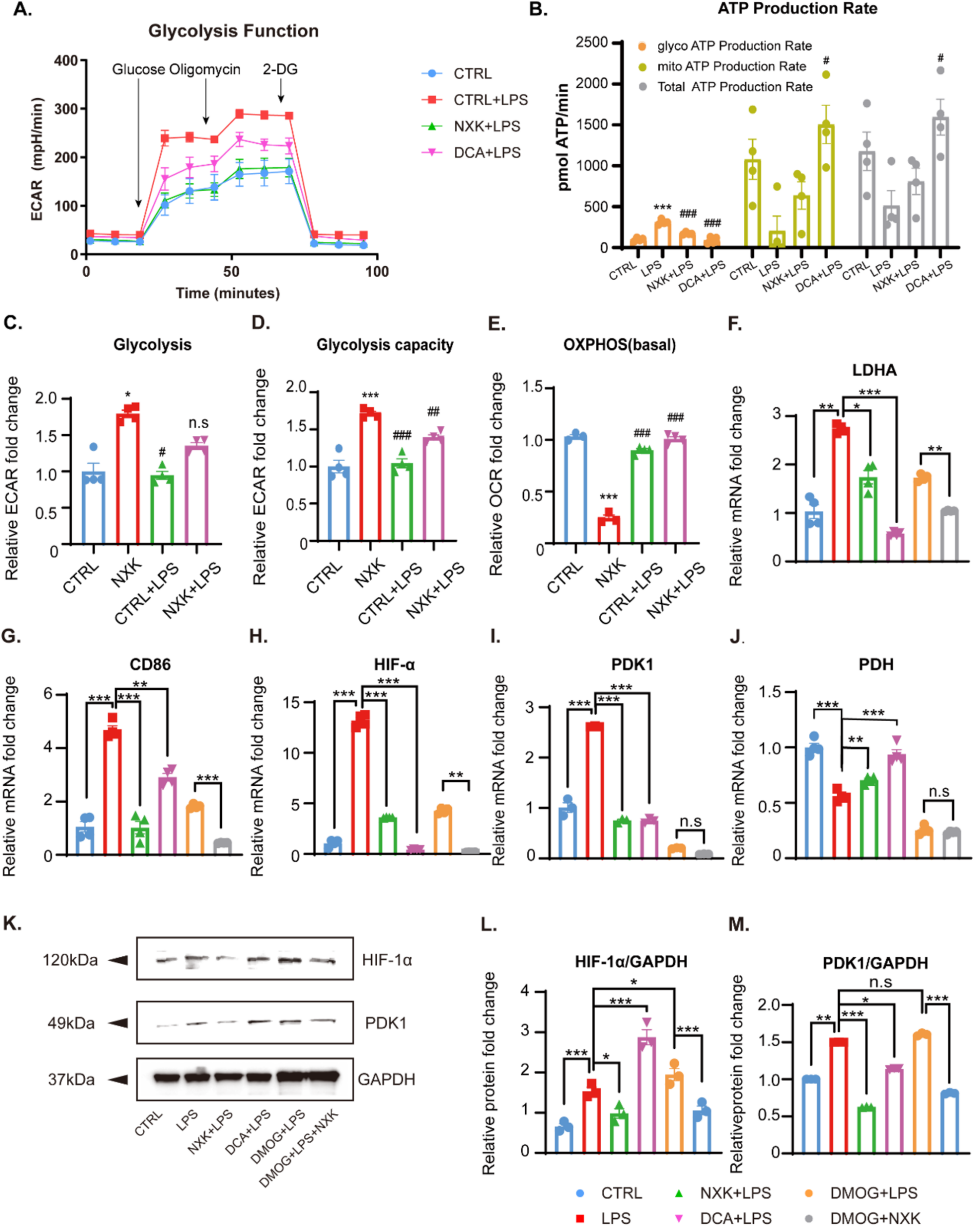
NXK regulates the energy metabolism of macrophages via the HIF-1α/PDK1 axis. **A** Representative curves of glycolysis function. **B** The real-time different types of ATP production rate (n = 4). **C**–**E** Glycolysis, glycolysis capacity, and basal OXPHOS were quantified (n ≥ 3). **F**–**J** The mRNA expression levels of cellular LDHA, CD86, HIF-1α, PDK1, PDH genes were quantified by qPCR (n ≥ 3). **K** Representative WB images of cellular HIF-1α and PDK1 in vitro. **L-M** Quantification of the cellular HIF-1α and PDK1 protein expression levels (n = 3). Values were shown as means ± SD, ^*^P < 0.05, ^**^P < 0.01, ^***^P < 0.001 vs the CTRL group, ^#^*P* < 0.05, ^##^*P* < 0.01, ^###^*P* < 0.001 vs the CTRL + LPS group, n.s, no significance (Panel **B**–**E**). P^*^ < 0.05, P^**^ < 0.01, P^***^ < 0.001, n.s, no significance (Panel **F**–**J** and **L**–**M**)

Then, to further verify that the HIF-1α/PDK1 axis plays a key role in the regulation of macrophage metabolic reprogramming by NXK, we introduced the HIF-1α agonist DMOG (5 mM) into the medium and used the PDK1 antagonist DCA (100 μM) as a positive control before measuring glycolysis levels under different treatments. Macrophages treated with NXK and stimulated by LPS showed lower glycolytic levels, glycolytic potential, and glycolytic ATP production rates, while NXK partially restored cellular OXPHOS levels (Fig. 7A–E), consistent with the results in Fig. 5. Furthermore, the mRNA expression levels of the key glycolytic gene LDHA, the M1 marker CD86, HIF-1α, and PDK1 were decreased (Fig. 7F–I), while the constraint on PDH was relieved (Fig. 7J). The efficacy of DCA in inhibiting LPS-induced overall glycolysis levels in macrophages, restoring cellular mitochondrial OXPHOS function, and regulating the HIF-1α/PDK1 axis is similar to that of NXK. These findings indicate that both NXK and the PDK1-specific inhibitor DCA led to a significant reduction in glycolysis induced by the massive activation of pro-inflammatory M1-like macrophages stimulated by LPS and DMOG. Moreover, DMOG, an HIF pathway activator, has been shown to increase HIF stability, after which the HIF complex is translocated to the nucleus to bind to HRE and regulate the expression of specific downstream factors such as PDK1 [11]. Surprisingly, we found that NXK could partially block HIF-1α transcription or translation of HIF-1α before exerting a repressive effect on PDK1 protein expression, implying a role for NXK in the HIF-1α/PDK1 axis Fig. 7H, I, K–M). Taken together, our findings suggest that NXK regulates the differentiation of macrophage phenotype through macrophage metabolism via the HIF-1α/PDK1 axis.

## Discussion

Macrophage polarization plays a pivotal role in mediating the immune-inflammatory response post-MI and is tightly linked to cardiac remodeling and congestive heart failure (CHF). Our study identifies an upsurge in M1-like macrophages and a decline in M2 markers post-MI, accompanied by increased glycolysis and suppressed OXPHOS in LPS-stimulated M1-like macrophages. NXK treatment significantly diminishes M1-like macrophages, reduces glycolysis, and reinstates mitochondrial OXPHOS. Further research revealed that NXK inhibits M1-like macrophage polarization and glycolysis via the HIF-1α/PDK1 axis, fostering M2-like macrophages and OXPHOS, effectively restoring equilibrium in the M1/M2 polarization ratio. This balance alleviates inflammatory damage, enhances cardiac function, and forestalls VR, emphasizing the critical role of the HIF-1α/PDK1 axis in mediating NXK's anti-inflammatory and energy metabolism-regulating effects in MI treatment.

In the early stages following MI, a significant influx of bone marrow-derived monocytes migrates to the infarcted area, subsequently differentiating into diverse macrophage subtypes. M2 macrophages, characterized by the expression of factors promoting cell proliferation and angiogenesis, contribute to immune regulation, anti-inflammatory processes, and tissue repair. However, dysregulation of M2 macrophages can lead to abnormal ECM proliferation and myocardial fibrosis, worsening cardiac remodeling. Maintaining a balanced M1/M2 macrophage ratio is paramount in effectively managing the post-MI inflammatory response. Our study shows that post-MI, M1-like macrophages are overactivated, while M2 macrophages decrease. NXK treatment restores this balance, reducing M1 markers and inflammatory factors while concurrently increasing M2 markers. This shift promotes tissue repair and mitigates inflammatory damage. This suggests that NXK possesses potential to modulate macrophage polarization, offering promising prospects in the prevention and management of MI.

Advancements in immunological research have shed light on the intricate link between macrophage polarization states and their metabolic profiles, influencing their trajectory. Specifically, M1 macrophages lean towards glycolysis, while M2 macrophages prefer OXPHOS. Our study highlights a metabolic shift from OXPHOS to glycolysis in LPS-stimulated macrophages, marked by reduced PDH activity and TCA cycle influx. This underscores the impact of metabolic reprogramming in polarization. Furthermore, research indicates that the HIF-1α/PDK1 axis promotes macrophage migratory capacity and systemic inflammation, and inhibiting glycolysis through this axis can counteract the progression of ischemia/reperfusion (I/R) injury and fibrosis [2, 6, 17]. The HIF-1α/PDK1 axis can affect the TCA cycle by regulating PDH and actively suppresses mitochondrial OXPHOS by upregulating PDK1, which phosphorylates and inhibits PDH, leading to reduced TCA cycle flux and OXPHOS inhibition [17]. Notably, NXK down-regulates LPS-induced glycolysis and inflammatory markers via the HIF-1α/PDK1 axis in the context of MI. Additionally, NXK reduces LPS-induced ROS production, potentially mitigating mitochondrial oxidative stress damage and releasing PHD activity. By lowering ROS levels, it is possible to reduce the stability of the HIF-1α subunit and thus the HIF signaling [8]. These findings suggest that NXK can modulate macrophage energy metabolism through the HIF-1α/PDK1 pathway, balancing M1/M2 macrophage polarization and aiding inflammation regulation and wound repair post-MI, ultimately alleviating cardiac injury.

In our previous study [3], ultra-high-performance liquid chromatography-mass spectrometry (UHPLC-MS) was utilized to uncover the bioactive constituents in the NXK formulation. Network analysis identified 21 bioactive ingredients. Subsequent research consistently supported the efficacy of these NXK ingredients in mitigating inflammation, safeguarding against I/R injury, preventing apoptosis, and regulating energy metabolism, aligning with NXK's recognized cardioprotective role.

**Fig. 8:**
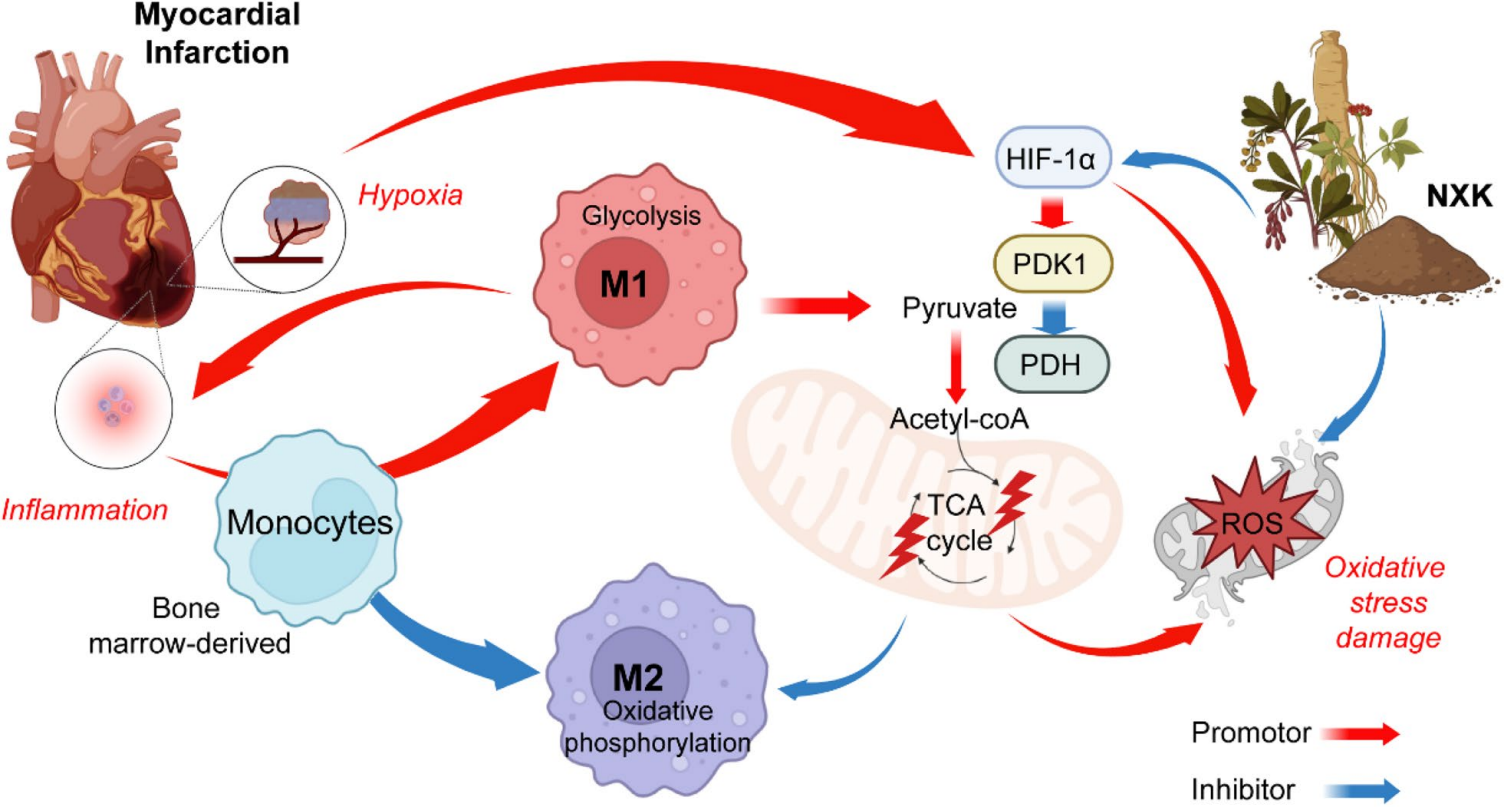
NXK orchestrates macrophage polarization by reprogramming energy metabolism based on HIF-1α/PDK1 axis. In the early stages of MI, M1 macrophages are heavily recruited and localized in a relatively hypoxic state, which activates HIF-1α. HIF-1α can participate in macrophage migration and polarization through PDK1-induced active glycolysis, which reduces the flux of TCA circulation by inactivating PDH. These suggest NXK can participate in macrophage metabolic reprogramming by inhibiting the HIF-1α/PDK1 axis, thereby regulating macrophage polarization, affecting the ratio of M1/M2 during post-MI repair, and improves cardiac remodeling

Our research unveiled NXK's ability to diminish ROS production, thereby stabilizing HIF-1α, reducing glycolysis and promoting OXPHOS (Fig. 8). This study emphasizes the impact of metabolic regulation on macrophage polarization, suggesting a potential correlation between metabolic reprogramming and phenotypic differentiation. These findings bear substantial implications for identifying novel targets to orchestrate macrophage polarization and metabolic reprogramming post-MI. This not only advances our understanding of underlying mechanisms but also paves the way for drug development, offering innovative prospects for treating MI and related conditions.

It's important to acknowledge the limitations of our study. Firstly, certain aspects of the precise mechanisms underlying the treatment of MI and specific details about the bioactive components within NXK's main ingredients remain unknown, necessitating further research for elucidation. Secondly, in our study, we did not involve genetic modifications of the HIF-1α and PDK1 genes or the use of inhibitors in the in vivo experiments. However, future research endeavors will explore these approaches to gain valuable insights into the mechanisms and enhance our understanding of how NXK functions in the context of MI treatment.

## Conclusion

Our study demonstrates that NXK treatment effectively preserves cardiac function after MI by modulating the HIF-1α/PDK1 axis. This modulation regulates the shift in macrophage phenotype from pro-inflammatory to anti-inflammatory, thereby reducing cardiac inflammatory injury and preventing adverse cardiac remodeling. The significant role of this axis in driving the macrophage phenotype shift, likely through its regulatory influence on macrophage energy metabolism, suggests that NXK holds therapeutic promise for MI patients by targeting a metabolic-inflammatory regulatory network. Further research to uncover the bioactive components and underlying mechanisms of action of this Chinese herbal formula is warranted.

### Supplementary Information

**Additional file 1: Figure S1.** The legend for gates-setting of flow cytometry in macrophages.

## Data Availability

Not applicable.

## Abbreviations

ANP: Atrial natriuretic peptide
BNP: Brain natriuretic peptide
CCL17: C-C motif chemokine 17
CCL12: C-C motif chemokine 12
CHF: Chronic heart failure
CK-MB: Creatine kinase muscle and brain isoenzym
COL1: Collagen types I
COL3: Collagen types III
cTn-I: Cardiac troponin I
DCA: Dichloroacetate
DMOG: Dimethyloxalylglycine
ECAR: Extracellular acidification rate
ECM: Extracellular matrix
GAPDH: Glyceraldehyde 3-phosphate dehydrogenase
HIF-1α: Hypoxia inducible factor-1α
HRE: Hypoxia response elements
IDH: Isocitrate dehydrogenase
IL-1β: Interleukin-1β
IL-6: Interleukin-6
iNOS: Inducible nitric oxide synthase
I/R: Ischemia/reperfusion
LDHA: Lactate dehydrogenase A
LPS: Lipopolysaccharide
LVAWd: Diastolic left ventricular anterior wall thickness
LVAWs: Systolic left ventricular anterior wall thickness
LVEF: Left ventricular ejection fraction
LVEDV: Diastolic volume of left ventricle
LVESV: Systolic volume of left ventricle
LVFS: Left ventricular fractional shortening
LVPWd: Diastolic left ventricular posterior wall thickness
LVPWs: Systolic left ventricular posterior wall thickness
MI: Myocardial infarction
MMP: Matrix metalloproteinase
NXK: Nuanxinkang
OCR: Oxygen consumption rate
OXPHOS: Oxidative phosphorylation
SRC: Spare respiratory capacity
PDH: Pyruvate dehydrogenase
PDK1: Pyruvate dehydrogenase kinase 1
qPCR: Quantitative Real-Time PCR
Retnla: Resistin-like molecule alpha
ROS: Reactive oxygen species
SDH: Succinate dehydrogenase
TCA: Tricarboxylic acid
TGF-β: Transforming growth factor-β
TNF-α: Tumor necrosis factor-α
TTC: 2,3,5-Triphenyl tetrazolium chloride
VEGF: Vascular endothelial growth factor
VR: Ventricular remodeling
WB: Western blotting
