# Supplementary figures and images for: A simplified herbal decoction attenuates myocardial infarction by regulating macrophage metabolic reprogramming and phenotypic differentiation via modulation of the HIF-1α/PDK1 axis
Source: Chin Med. 2024 May 30;19:75. doi: 10.1186/s13020-024-00933-x (PMC11140944; doi:10.1186/s13020-024-00933-x)

**Figure S1 The legend for gates-setting of flow cytometry in macrophages.**

**
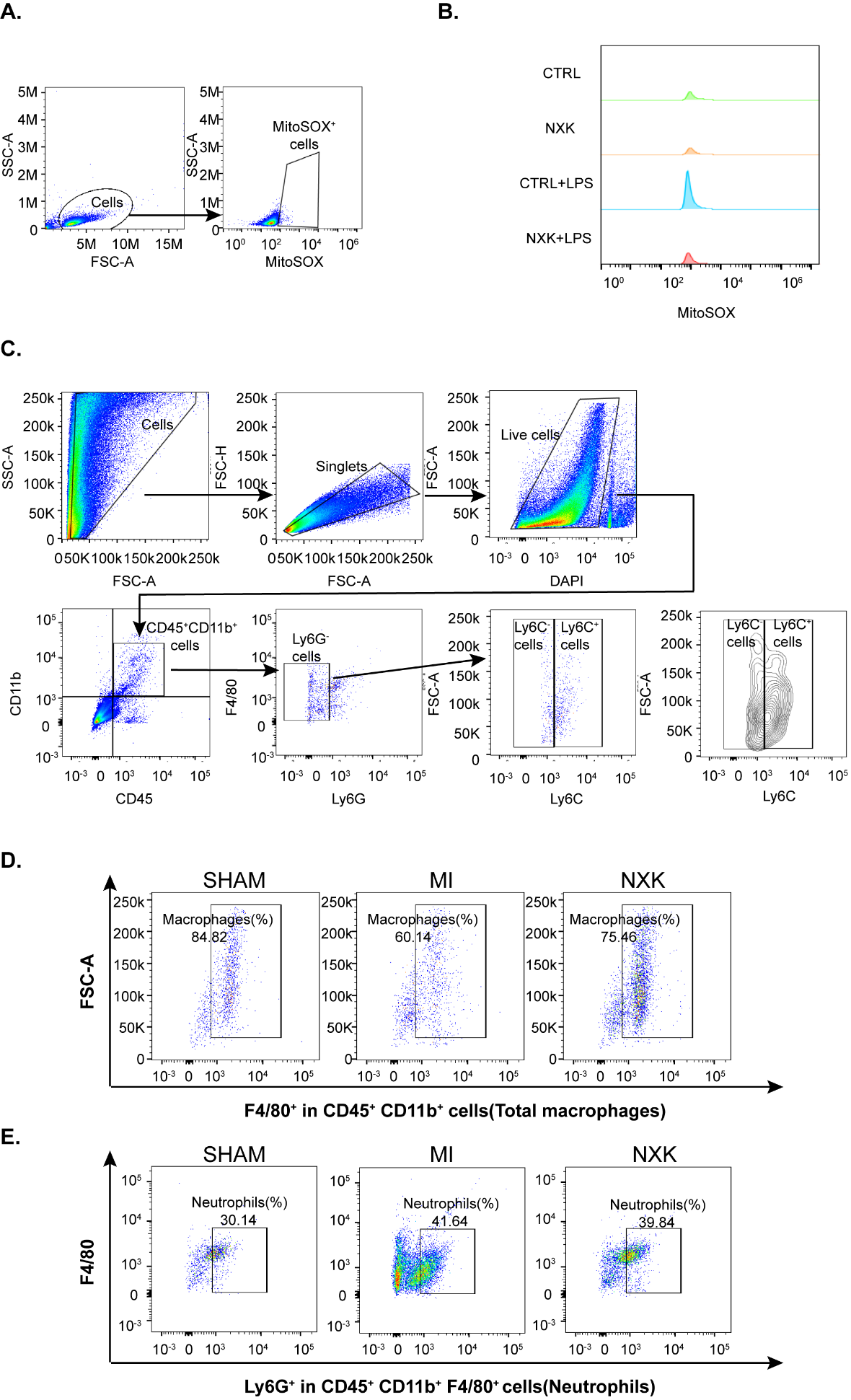
**

Supplement: Supplementary file 1 — Additional file 1: Figure S1. The legend for gates-setting of flow cytometry in macrophages. [file 13020_2024_933_MOESM1_ESM.docx]
